# Towards a practical *O*(*n*log*n*) phylogeny algorithm

**DOI:** 10.1186/1748-7188-7-32

**Published:** 2012-11-26

**Authors:** Jakub Truszkowski, Yanqi Hao, Daniel G Brown

**Affiliations:** 1 David R. Cheriton School of Computer Science, University of Waterloo, Waterloo ON N2L 3G1 Canada

**Keywords:** Phylogeny, Random walk, Quartet

## Abstract

Recently, we have identified a randomized quartet phylogeny algorithm that has *O*(*n*log*n*) runtime with high probability, which is asymptotically optimal. Our algorithm has high probability of returning the correct phylogeny when quartet errors are independent and occur with known probability, and when the algorithm uses a guide tree on *O*(loglog*n*) taxa that is correct with high probability. In practice, none of these assumptions is correct: quartet errors are positively correlated and occur with unknown probability, and the guide tree is often error prone. Here, we bring our work out of the purely theoretical setting. We present a variety of extensions which, while only slowing the algorithm down by a constant factor, make its performance nearly comparable to that of Neighbour Joining , which requires Θ(*n*^3^) runtime in existing implementations. Our results suggest a new direction for quartet-based phylogenetic reconstruction that may yield striking speed improvements at minimal accuracy cost. An early prototype implementation of our software is available at http://www.cs.uwaterloo.ca/jmtruszk/qtree.tar.gz.

## Background

Any useful phylogenetic reconstruction algorithm must use *Ω*(*n*log*n*) time to reconstruct a phylogeny of *n* taxa, because it takes that much time to write down a tree (see, *e.g.*, [1]). In practice, all commonly used algorithms take much longer runtimes. To optimize parsimony, likelihood or the least-squares objective is NP-hard, and the best distance-based methods require Θ(*n*^3^) runtime in the worst case for Neighbour Joining , or Θ(*n*^2^) time for UPGMA [2]. Typical quartet methods require *Ω*(*n*^4^) time, since they enumerate all quartets. Common heuristics for the problem run in quadratic time, and the recent program FastTree has a runtime of Θ(*n*^1.5^log*n*) in practice. Programs with such a subquadratic runtime are necessary: many projects currently desire trees on hundreds of thousands of taxa, and new projects such as metagenomics or sequencing individual cells inside a human may require creating trees from millions of sequences.

We have recently developed a quite different phylogenetic approach, based on quartet queries, which achieves the *O*(*n*log*n*) runtime lower bound, in expectation and with high probability, while giving probabilistic guarantees on the quality of its performance in a simple error model [3]. It also promises to return the correct topology with 1−*o*(1) probability. Our algorithm is randomized, so its runtime is a random variable, but its expectation is *O*(*n*log*n*) regardless of the true topology of the tree.

However, the probabilistic assumptions on which the program’s error analysis depends are not realistic: we require that quartet queries err both independently and with known probability. This presumption is false: for example, two quartet queries that include the same taxon are definitely not independent. Moreover, some quartets may have evidence of being of high quality, while others are not very good. Our initial description of our algorithm did not incorporate this evidence. Our algorithm also only places some taxa into the final tree; with high probability in the theoretical model, all taxa are placed, but in preliminary experiments, 40% of taxa were not present in the tree returned.

Here, we describe our work to bring our algorithm away from a purely theoretical approach to a substantially more practical, still extremely fast, phylogenetic reconstruction method. We describe a number of extensions to our method which increase both the fraction of taxa in the returned tree, which we call *coverage*, and the accuracy of the topology of the tree that is returned. As a result of these extensions, the coverage of our algorithm is now over 90%, and the accuracy of the tree that results approaches that of Neighbour Joining : see the experiments section. When combined with local search, our algorithm can produce trees on the full taxa set whose accuracy is almost identical to that of FastTree, while being substantially faster. This paper is an expanded version of our earlier conference publication [4].

Our methods show that quartet-based algorithms, properly used, can form the basis of an effective phylogenetic reconstruction method, in time that is both theoretically and practically faster than any commonly used approach for the problem.

## Background and Related work

Our algorithm is an *incremental* phylogeny algorithm: it starts with a guide tree on a small number of taxa, and then inserts each new taxon *s*_*i*_ into the phylogeny created by the first *i*−1 taxa, until all *n* taxa are inserted. We use a randomized balanced search tree structure to ensure that, with high probability, it requires *O*(log*i*) time to insert taxon *s*_*i*_.

### Definitions

A *phylogenyT* is an unrooted binary tree with *n* leaves in 1-to-1 correspondence with a set  of terminal taxa. Removing an internal node *v* from a phylogeny yields three subtrees, *t*_*i*_(*T*,*v*) for *i*=1,2,3. The tree *t*_*i*_(*T*,*v*) joined with its edge to *v* is the *child subtreec*_*i*_(*T*,*v*).

A *quartet* is a phylogeny of four taxa. A *quartet queryq*(*a*,*b*,*c*,*d*), returns one of three possible quartet topologies: *ab*|*cd*, *ac*|*bd* and *ad*|*bc*: in *ab*|*cd*, if we remove the internal edge, we disconnect {*a*,*b*} from {*c*,*d*}. In our work, we assume a quartet query can be done in *O*(1) time; we discuss how to perform such queries in Section “Quartets”.

A *node queryN*(*T*,*v*,*x*) for internal node *v* of phylogeny *T* and new taxon *x* is a quartet query *q*(*x*,*a*_1_,*a*_2_,*a*_3_), where *a*_*i*_ is a leaf of *T* in *t*_*i*_(*T*,*v*). Such a query identifies the *c*_*i*_(*T*,*v*) where taxon *x* belongs, if the returned quartet is correct.

### Summary of our previous work

Our algorithm incrementally adds each taxon to the growing tree, using quartet queries to direct the taxon to smaller and smaller parts of the tree. By using a structure that is balanced (with high probability), we ensure that each search requires logarithmic time, giving the asymptotically optimal runtime bound.

**Figure 1:**
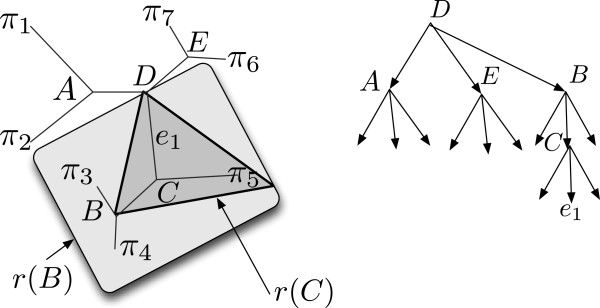
**A search tree.** A phylogeny and a corresponding search tree. Internal nodes of the search tree correspond to subphylogenies: two, for *r*(*B*) and *r*(*C*), are indicated. Leaves of the search tree correspond to edges of the phylogeny.

We direct the queries using a rooted ternary search tree structure (Figure 1). Each internal node *v* in our search tree corresponds to a pair: a contiguous region *r*(*v*) of the phylogeny, and a node *s*(*v*) within that region. Each leaf *v* of the search tree corresponds to an edge *r*(*v*) in the phylogeny. For an internal node *v* of the search tree, its three children correspond to each of the three *t*_*i*_(*r*(*v*),*s*(*v*)) subtrees. If node queries are always correct, then asking the node query (*T*,*s*(*v*),*x*) will identify which of the *t*_*i*_(*r*(*v*),*s*(*v*)) subtrees the taxon *x* belongs in, and a traversal through the search tree to a leaf will identify which edge of *T* is the one where the new taxon *x* belongs.

Quartet errors are common, which would lead to errors in this simple algorithm. To allow for their existence, we incorporate a random walk method: rather than requiring that we only move down the search tree, we allow for the possibility that we backtrack. Specifically, at node *v* in the search tree, we ask up to two node queries, corresponding to the boundaries of the current region of the phylogeny; if either of them gives evidence that the new taxon does not belong in *r*(*v*), we move to the parent of the current node in the search tree. If they are consistent with *r*(*v*), we ask a node query at (*T*,*s*(*v*),*x*), and that identifies which child of *v* we go to in the search tree.

Assuming that there is a bias toward going in the correct direction, and that query errors are independent, there is a steady push toward winding up at the correct node in the search tree: the random walk has positive drift, so it moves at an expected constant speed toward the true goal. If we are at a leaf, then we continue the algorithm, but each query that pushes us to stay at the chosen leaf increments a counter and each query that pushes us away from the leaf decrements the counter. We only move up from the leaf in the search tree when the counter has value zero.

This random walk method is inspired by an approach for sorting a list where comparisons are correct with probability greater than 1/2, but err independently with known probability [5]. Our adaptation of the approach increases the number of queries required to insert each new taxon by a constant factor (depending on error probability) over simply descending the search tree. It guarantees that each new taxon is placed in the correct location with 1−*o*(1/*n*) probability.

Further, the algorithm runs in *O*(*n*log*n*) runtime both in expectation and with high probability, since if we use a uniformly-chosen permutation, the search tree remains balanced with high probability. For full details, we refer the reader to our original paper [3].

One key technical detail is that we must ensure that the algorithm never runs out of queries to ask: each new quartet query must be distinct from all the previous ones. This is achieved by first constructing a “guide tree” phylogeny for a large enough subset of taxa. We then build a search tree for the guide tree and insert the remaining taxa into the search tree in random order. Additionally, we guarantee that the guide tree is accurate; in our original paper [3], we show this holds with probability 1−*o*(1) when the tree is the maximum quartet consistency tree of a set of loglog*n*taxa.

In practice, larger guide trees are more likely to be accurate, thus improving the accuracy of the whole algorithm. For guide trees reconstructed using Neighbour Joining, we recommend using guide trees on *cn*^1/3^taxa, for a small enough value of *c*. This ensures that the time spent on reconstructing the guide tree is negligible compared to the running time of the main algorithm, as reconstructing the guide tree takes linear time.

### Quartets

There are several ways of inferring the subtopology of a phylogeny corresponding to just four taxa, {*W*,*X*,*Y*,*Z*}. Perhaps the most natural is to compute an estimate *d*_*i*,*j*_for the pairwise distance between all pairs from the four taxa, and then use ordinary least-squares estimation to compute the additive distance matrix closest to the inferred distance matrix, and return the quartet corresponding to the structure of that distance matrix. This is equivalent to comparing *d*_*W*,*X*_ + *d*_*Y*,*Z*_,*d*_*W*,*Y*_ + *d*_*X*,*Z*_ and *d*_*W*,*Z*_ + *d*_*X*,*Y*_; if the smallest of the three values is the first, we infer topology *WX*|*YZ*, if the second is smallest, we infer *WY*|*XZ*, and if the third is smallest, we infer *WZ*|*XY*.

In practice, quartet topology estimation is notably challenging. If the middle edge of the true quartet is short and the other four edges are long, uncertainty in estimating the four pairwise distances that cross the middle edge may dominate the actual length of the edge, making quartets whose true topologies are hard to infer. In a balanced phylogeny where each edge is of constant length, a constant fraction of all quartets will have this problem.

As the traversal of the search tree progresses, the region of the tree that is under search shrinks, so quartets will correspond to smaller total lengths, meaning that the fraction of the total edge length of the quartet that is falling on the middle edge will increase; this suggests that quartet errors may be rarer at lower levels of the search tree, as we discuss in the next section.

Other methods to infer quartets include the ordinal quartet method [6], weighted least-squares, and using maximum-likelihood methods. Our algorithm assumes all of these methods operate in *O*(1) time on a quartet, as our runtime does not depend on the phylogenetic source data, whether it is biological sequences, an alignment of such sequences, or microsatellite or other data with phylogenetic signal. Indeed, our algorithm works in any scenario in which quartets can be successful with sufficiently high probability.

### Other fast phylogenetic algorithms

The tree that optimizes the Neighbour Joining objective is not known to be producible in *o*(*n*^3^) time in worst case. However, heuristics for this problem have been a subject of much research; Wheeler [7] gives an exact Neighbour Joining implementation, using clever data structures, which seems to require sub-cubic runtime in typical instances, while other authors [8,9] have given heuristic algorithms that operate in *O*(*n*^2^) time and that typically run in $O(n\sqrt{n}\log n)$ time, but do not guarantee to optimize the Neighbour Joining objective. The UPGMA objective can be optimized in *O*(*n*^2^) runtime [2]. Desper and Gascuel [10] give a heuristic for the minimum-evolution objective that runs in *O*(*n*^2^log*n*) for most trees.

Erdös and co-authors have shown that the short quartet method can solve the phylogenetic inference problem in *O*(*n*^2^polylog *n*) time on most trees, on sufficiently long sequences, and assuming standard Markov models of evolution [1]; Csűrös gives a practical algorithm that has similar guarantees [11]. There is also a developing literature on sequence length requirements for various phylogeny algorithms, and on identifying parts of the tree that can be reconstructed from a given alignment [12].

Sub-quadratic phylogeny methods are quite rare: King *et al.*[13] give an $O(n^{2}\frac{\log\log n}{\log n})$-time algorithm that is not widely used. When data are error free, our algorithm is not the first *O*(*n*log*n*) algorithm, as Kannan *et al.*[14] have given a rooted-triple algorithm with this runtime.

A more distressing lower bound, also in the same paper of King *et al.*, shows that any distance-based method requires $\Omega (\frac{n^{2}}{\log n})$ time to reconstruct correct trees where a simple Markov model of evolution is assumed, and distances are inferred using standard techniques, assuming sequences are quite short.

### Quality measures

We present two different quality measures: Robinson-Foulds quality and quartet quality. Given a ground-truth topology for a set of taxa and a second topology on the same taxa, the Robinson-Foulds quality is the fraction of the non-trivial splits (internal edges) of one topology found in the other. The quartet quality (see, *e.g.*, [15,16]) is the fraction of the n4 quartets that have the same result in both tree topologies. The Robinson-Foulds measure is fragile: a single misplaced taxon can ruin all of the splits of the tree. By contrast, the quartet distance is robust to individual errors: even a randomly-placed taxon will probably have the correct topology in one-third of its quartets, as there are only three choices for the topology.

## Extensions to improve performance

Our basic algorithm suffers from a number of issues that greatly limit its practical performance. The accuracy of quartet inference is often very low, particularly for quartets asked at the top nodes of the search tree; when running the basic algorithm on the COG840 data set [9], we found that only 66% of quartets were inferred correctly, with only 50% of correct inferences at the root of the search tree. In practice, quartet inference errors are also not independent, which may cause the random walk to “drift away” from the optimal placement of the taxon either to an incorrect place or to drift in the higher nodes of the search tree, leading to reduced coverage. In this section, we discuss a number of improvements to the algorithm to address these problems.

### Quartet weights

The basic algorithm considers all quartets to be equally reliable. In practice, some quartets are more likely to be correct than others. Assigning equal weight to all quartets can lead to serious errors due to many erroneous quartets. Various quartet phylogeny algorithms estimate the reliability of inferred quartet topologies based on their likelihood scores [17-19] or distances between taxa (*e.g.*[20]).

We assign quartet weights based on the inferred middle edge length normalized by the the sum of all the edge lengths of the quartet. For a quartet with external edge lengths *a,b,c,d* and an internal edge length *e*, this becomes *w*=*e*/(*a* + *b* + *c* + *d* + *e*). The intuition behind this idea is that if the inferred middle edge is long compared to the distances between taxa, then the inferred quartet topology is less likely to have arisen from errors in the distance estimates. The edge lengths are inferred using ordinary least squares, which was dictated by the speed of this approach.

We note that this weighing scheme is somewhat different than other commonly used schemes. Most quartet algorithms use likelihood weights as opposed to distances. Snir *et al.*[20] use the topological diameter of the quartet in a preliminary tree constructed by Neighbor Joining.

To incorporate the reliability information into the algorithm, we ask *k* quartet queries at each node query and vote according to the weights. We have tested two voting schemes. In the weighted-majority scheme, the weights of each quartet pointing in the same direction are added and the direction with the highest vote total is chosen. In the winner-takes-all scheme, the direction is chosen according to the quartet query with the highest weight from the *k* used. We found that the weighted-majority voting scheme results in better accuracy according to the quartet measure, while the winner-takes-all approach yields higher accuracy according to the Robinson-Foulds measure (see the experiments section).

### Biased choice of quartets

Many authors have suggested that large distances between taxa lower the accuracy of quartet inference [1,20]. While our algorithm is forced to ask long quartets at the top of the search tree, biasing the choice towards shorter quartets at the lower nodes of the search tree might reduce the number of unreliable quartets. When asking a node query at node *y* of the search tree, we require that the representatives for each quartet query are contained in a subtree *r*(*y*^∗^) associated with a node *y*^∗^that is at most *d* levels above *y* in the search tree. The value *d* is chosen for each *y* so that the number of possible representatives in each direction is at least 20. Note that this does not change the behaviour of the algorithm in the upper parts of the search tree, since the subtrees associated with the upper nodes are typically much larger. This heuristic is used in all experiments.

### Multiple insertion rounds

Due to the ambiguity in inferred quartets, the random walk will often terminate at an internal node in the search tree, resulting in the taxon not being inserted into the phylogeny. After the algorithm has attempted to insert every taxon in the search tree, we try reinserting the taxa that did not make it to a leaf in the first round. We have two such rounds of reinsertions.

### Confidence threshold

Incorrectly inserting a taxon can prevent subsequent taxa from being inserted in the correct place. To mitigate this, we only add a new taxon to the phylogeny if it has spent more than the *ℓ*=30 last steps of the random walk at the current leaf.

### Repeating the random walk

To improve our confidence in the placement of new taxa, we ran the random walk two times for each inserted taxon. The taxon was then inserted only if both walks terminated at the same leaf node.

## As-yet unsuccessful ideas

Several natural methods for improving our algorithm’s performance have not yet been successful. We note these extensions here to document the challenging process of improving a theoretical prototype into a useful method for phylogenetic inference.

Increasing the number of quartet queries without weighting them improved the fraction of taxa inserted, but decreased the accuracy. This emphasizes the need for weighing quartets.

We have tried various other methods of estimating the reliability of quartets. Earlier work on short quartet methods [1,20] suggested using the diameter of the quartet for estimating its reliability. Contrary to our expectations, this approach did not provide satisfactory results. This may be because the diameter of the inferred quartets varies widely between different levels of the search tree. Quartets inferred near the root of the search tree tend to have large distances between taxa, whereas quartets inferred in the deeper parts of the search tree are shorter since they only span a small fraction of the overall tree. Using least squares fit of the distances to the quartet topology to weigh the quartets also did not seem to improve the accuracy of node queries. Using weighted least squares did not improve the accuracy of quartet inferences.

We have tried to find a good starting point for the random walk by using profile search. For each of the top log*n* nodes of the search tree, we constructed a profile of sequences in the subtree associated with that search tree node. Before starting the random walk for the new taxon, we aligned its sequence to each profile and started the random walk from the subtree whose profile gave the highest alignment score. This did not have a significant effect on the results, perhaps because of the large diversity of sequences within the top subtrees.

## Experiments

We have evaluated our algorithm on several simulated data sets. The simulated data sets are taken from Price *et al.*[9] who used them to evaluate their heuristic program FastTree, which has *O*(*n*^1.5^log*n*) runtime on typical instances. These data sets were generated by taking real protein alignments and their maximum likelihood trees, and then simulating evolution of sites along that phylogenetic tree, treating it as ground truth. The evolutionary rates varied across sites.

In most experiments, the initial guide tree was created using Neighbor Joining on a randomly chosen subset of 200 taxa. For protein sequences, our algorithm uses ScoreDist [21] to estimate distances. FastTree uses a similar, but distinct, distance measure [9]. For nucleotide sequences, both algorithms use the standard log-corrected Jukes-Cantor distance. For a fair comparison of accuracies and running times, we only compare against the initial Neighbor Joining phase of FastTree. FastTree then performs nearest neighbour interchanges to improve the quality of the resultant tree, which takes up much of its runtime; we have not incorporated these into our algorithm, either, so we compare against the initial phase of FastTree for consistency of comparison.

In the first experiment, we assessed how various improvements discussed in the previous section changed the performance of the algorithm. We ran each version of the algorithm 100 times on the COG840 data set. The results are given in Table 1.

**Table 1:** The effect of heuristics on the performance of the algorithm

**method**	**% taxa**	**RF**
	**inserted**	**accuracy**
basic RW+NJ guide tree	56.3 ± 2.4	46.3 ± 4.6
basic RW+true guide tree	57.3 ± 2.1	49.4 ± 5.0
(not feasible in practice)		
5 quartets per node query, UM	76.4 ± 2.0	41.0 ± 3.8
5 quartets, WM	85.6 ± 1.6	48.6 ± 3.7
5 quartets, WM, 2E	95.4 ± 1.0	45.5 ± 3.4
5 quartets, WM, CT, 2E	84.1 ± 1.8	57.4 ± 3.5
5 quartets, WM, re-running the RW, 2E	78.8 ± 2.4	59.5 ± 3.7
5 quartets, WTA, CT, 2E	80.2 ± 2.1	62.3 ± 2.9
20 quartets, WTA, CT, 2E	92.1 ± 1.4	60.8 ± 2.9
NJ	n/a	62.6

Shown are results for the COG840 data set with 1250 taxa. We show our algorithm’s performance in various settings, and compare it to Neighbor Joining. We report accuracies using the Robinson-Foulds measure. Our algorithm places approximately 80% − 90% of taxa with accuracy around 60%. We ran each version of the algorithm 100 times. In all cases, the guide tree is on 200 taxa; except in the second line of the table, this was generated with Neighbor Joining, and had RF accuracy of 50% ± 3%. Three voting schemes were used in the experiments: unweighted majority (UM), weighted majority (WM), and winner-takes-all (WTA). In some experiments, we also added 2 additional rounds of insertions (2E), and a confidence threshold for insertion (CT).

Overall, the improvements to the algorithm boosted the RF accuracy from 46% to around 60%, and increased the proportion of inserted taxa from 56% to 82-95%, depending on the settings. The biggest gains were achieved by introducing quartet weights and a confidence threshold. Multiple rounds of insertions increased the coverage of the taxa set. Increasing the number of quartets and running the random walk twice increased the accuracy, but at the cost of increased running time. It should be noted, however, that the increase in running time was not directly proportional to the number of quartets per query: one run of the algorithm for 5 quartets per query takes around 11 seconds, compared to just under 20 seconds for 20 quartets per query. This is because the running time is dominated by computing the distances, which we save and reuse in the subsequent quartet queries.

In general, WTA voting tended to produce trees with higher RF accuracy than weighted-majority voting. In contrast, weighted-majority voting resulted in higher quartet accuracy, as we shall see in the next experiment.

We see that there is a trade-off between the proportion of the inserted taxa and the RF accuracy. This is not surprising since incorrectly inserting a hard-to-place taxon into the phylogeny can cause many splits to be incorrect.

The accuracy of guide trees was considerably lower than that of the NJ tree for the full data set. To investigate the impact of errors in the guide tree on the accuracy of the algorithm, we ran the algorithm starting from a guide tree consistent with the true phylogeny. Contrary to our expectations, errors in the guide tree have little impact on the accuracy; substituting the NJ guide tree with the true one improved the accuracy by 2 to 3 percent, depending on the version of the algorithm.

In the second experiment, we evaluated the algorithm on three unrelated data sets having 250, 1250, and 5000 taxa, respectively. For each data set, we ran the algorithm in four different configurations, using two voting schemes and varying the number of quartet queries per node query. The size of the guide tree was 200 except for the 250 taxon data set, where it was set to 100. The results are shown in Table 2.

**Table 2:** Comparison of different algorithms

					**data set**				
	**COG1011**			**COG840**			**COG1028**		
	**250 sequences**			**1250 sequences**			**5000 sequences**		
**method**	**% taxa**	**RF**	**QA**	**% taxa**	**RF**	**QA**	**% taxa**	**RF**	**QA**
weighted majority, 5 quartets	88.5	66.2	72.8	84.1	57.4	85.8	74.4	51.4	59.5
WTA-vote, 5 quartets	86.0	69.4	70.8	80.2	62.3	85.4	70.1	57.0	59.6
weighted majority, 20 quartets	95.6	60.4	69.9	96.4	50.6	83.9	94.3	41.1	56.5
WTA-vote, 20 quartets	94.0	69.4	73.4	92.1	60.8	83.1	89.7	57.6	55.3
NJ	100	73.6	70.0	100	62.6	88.0	100	73.0	66.3
FastTree (NJ phase only)	100	69.7	85.9	100	61.0	86.6	100	73.6	66.4
weighted majority, 5 quartets, force all taxa	100	59.0	69.7	100	48.7	80.8	100	37.3	52.4

Performance of the random walk algorithm on synthetic alignments. The size of the guide tree was 200 except for the 250 taxon data set, where it was set to 100. The average RF accuracy of the guide trees was 65,50, and 46% for the 250,1250, and 5000-taxon data sets, respectively. The average quartet accuracies for the guide trees were 73,83 and 55%. When all taxa are forced into the random walk tree (see text), the RF accuracy decreases by 7 − 14%, depending on the data set. All random walk runs use the confidence threshold heuristic and two additional rounds of insertions.

We see that the weighted-majority voting scheme tends to produce trees with higher quartet accuracy, whereas the winner-takes-all voting scheme yields higher RF accuracy. In general, we see that the two measures are very different: an algorithm can have relatively good performance according to one while being considerably worse in the other, though the difference between voting schemes tends to be more pronounced for RF accuracy. In most cases, the accuracy of the random walk algorithm is lower than the accuracy of Neighbor Joining.

In the next experiment, we measured the running time of our algorithm on large data sets. For this experiment, we took a large simulated nucleotide alignment based on a real 16S alignment and compared the runtimes of the initial phase of FastTree and our algorithm. We ran the algorithms on the full alignment and on two smaller alignments created by randomly sampling 20000 and 40000 sequences from the large alignment. All running times were measured on a standard desktop computer with an AMD 7750 Dual Core 2712MHz processor and 4 GB RAM. In all cases, our algorithm runs faster, with the relative speedup increasing with the size of the data set; Table 3 shows the running times. Table 4 shows the accuracies obtained in these runs. In contrast to the previous experiment, our algorithm achieves higher accuracy than FastTree.

**Table 3:** Running times on large data sets

	**# of sequences**		
	**20,000**	**40,000**	**78,132**
weighted majority, 5 quartets	6m 41s	15m 52s	34m
FastTree (NJ phase only)	13m 52s	41m 15s	116m

Running times of the random walk algorithm compared to FastTree. We used the huge.1 alignment from the original FastTree paper [9]. Smaller data sets were created by choosing a random subset of sequences from the large alignment. Our algorithm runs 2.1 to 3.4 times faster than FastTree on these very large data sets.

**Table 4:** Accuracies on large data sets

		**# of sequences**	
	**20,000**	**40,000**	**78,132**
weighted majority, 5 quartets	80.8 ±1.1	78.9 ±1.4	80.8 ±1.1
weighted majority, 5 quartets, all taxa forced	79.9 ±1.1	77.8 ±1.4	75.3 ±1.6
weighted majority, 5 quartets+local search	96.1	94.4	92.8
weighted majority, 5 quartets, all taxa forced+local search	95.8	93.8	92.0
FastTree (NJ phase only)	62.9	58.1	52.2
FastTree + local search	95.8	93.8	92.0

Robinson-Foulds accuracies of FastTree and the random walk algorithm for the huge.1 data set [9]. The figures for the random walk algorithm represent the average accuracy over 10 runs of the algorithm, together with empirical standard deviations. We used a confidence threshold, with two additional rounds of insertions. The average taxon coverage for weighted majority was 98.6, 98.6, and 98.0 per cent for the 20,000, 40,000, and 78,132 taxa alignments, respectively. After applying local search, the variance between the runs of the random walk algorithm is negligible.

On the data set with 20000 sequences, the QuickTree implementation of Neighbour Joining [22] took over 360 minutes to complete and produced a tree almost identical to the one produced by FastTree, with over 99% of splits agreeing between the two trees. We were not able to run Neighbour Joining on larger data sets due to QuickTree’s memory consumption.

To investigate the differing relative performance of our algorithm and FastTree on different data sets, we generated more data sets under varying conditions. When designing this series of experiments, we wanted to test two hypotheses: first, that our algorithm is more accurate than FastTree when sequences of sufficient length are available; second, that our algorithm is more accurate when the branch lengths are short. Both of these are consistent with the differences between the COG data sets used in the first experiment and the 16S data sets used in the second experiment.

We simulated 10 trees on 20000 taxa from the pure-birth process. We then multiplied the length of each branch by a factor chosen uniformly at random from interval [0.5,2] to deviate the trees from ultrametricity. This methodology follows the previous work of Liu *et al.*[23]. We then scaled the branch lengths by several constant factors. For each choice of tree and scaling factor, we generated nucleotide alignments whose length varied between 250 and 4000 positions. The sequences were simulated from the Jukes-Cantor model, with variable evolutionary rates across sites drawn from the exponential distribution. No indels were introduced in the simulation. We used *rose*[24] to generate the sequences and *r8s*[25] to generate the trees.

**Figure 2:**
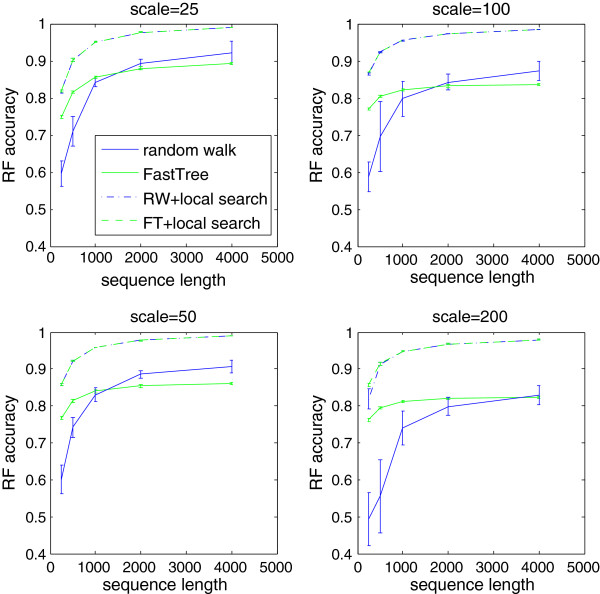
**Comparison of the random walk algorithm and FastTree.** The performance of the random walk algorithm and FastTree as a function of the length of the sequences. The four graphs represent the performance on 10 tree topologies with branch lengths scaled by constant factors 25,50,100, and 200. In all cases, the random walk algorithm compares increasingly favourably with FastTree as the sequence length increases. After applying local search, the differences between the average accuracies of the two methods are less than 1% for all the settings except the shortest sequences in the data set scaled by 200, where trees obtained from FastTree are 3.8% more accurate. The average taxon coverage for random walk trees was 97.3%, with only three experimental settings yielding coverage below 95%. Missing taxa were inserted into random walk trees before applying local search.

We see that the accuracy of both algorithms improves as more sequence data is available. However, the accuracy of the random walk algorithm improves faster than that of FastTree as sequences get longer. For trees with shorter branches, the advantage of the random walk algorithm is visible for shorter sequence lengths. Even for long sequences, the advantage of the random walk algorithm over FastTree was not as large as on the simulated 16S data set. Figure 2 shows the performance of the two algorithms in various conditions. The average taxon coverage for random walk trees was 97.3%, with only three experimental settings yielding coverage below 95%. Unsurprisingly, coverage was lower for short sequences and long branches.

### The impact of local search

FastTree uses local search to improve the accuracy of the Neighbour Joining tree. The improvement can be quite dramatic: for the huge.1.20000 alignment, local search improved the accuracy from 62% to 96%.

We ran FastTree’s local search procedure starting from trees obtained from our algorithm and the Neighbour Joining phase of FastTree for the tree sets used in the previous experiment. We inserted the remaining taxa into the random walk trees by running the random walk followed by a simple descent down the search tree. We then ran local search on the resultant trees. The results are shown in Figure 2. Despite huge differences in the accuracy of starting trees for some data sets, the trees improved by local search have very similar accuracies. This suggests that our algorithm can be combined with the local search phase of FastTree to produce trees that are very similar to FastTree, in less runtime. We also obtained similar results for the huge.1 data set (see Table 4).

The quality of the starting tree did not have much impact on the running time of the local search procedure. For all datasets we investigated, the differences between the two runs of local search were less than 15% of the overall running time. Table 5 shows the running times for the huge.1 data set.

**Table 5:** Running times on large data sets

		**# of sequences**	
	**20,000**	**40,000**	**78,132**
weighted majority, 5 quartets+local search	20m 40s (27m 21s)	41m 33s (57m 25s)	79m (113m)
FastTree + local search	21m 27s (35m 19s)	42m 29s (83m 44s)	80m (196m)

Running times of the local search procedure of FastTree applied to trees produced by our algorithm and the Neighbour Joining phase of FastTree on the huge.1 data set. Total runtimes, including the time required to produce the initial tree, are shown in brackets.

Trees produced by running local search on incomplete random walk trees (without forcing) had very similar accuracies compared to those obtained from the full taxa set; for all data sets, the difference was at most 3%. In most cases, the differences in accuracy were less than 1%. Before local search, the loss of accuracy was more substantial, with the RF accuracy dropping by up to 10% when the remaining taxa were inserted into the random walk tree. In most cases, this negative effect is much more minor, averaging less than 2%.

## Aggregating information from many trees

Our random walk algorithm is not limited to quartets inferred from aligned sequences. It can also be used with other types of quartet queries, *e.g.* evaluating how many times a given quartet topology appears in a collection of phylogenetic trees. We investigated how our algorithm can be used to aggregate conflicting information from many phylogenetic analyses to create a single, more accurate tree. Such algorithms are known as *supertree methods* or *consensus methods*.

In the first experiment, we ran the random walk algorithm five times on the COG840 data set, generating five distinct trees. We then ran the algorithm for the sixth time, this time answering quartet queries based on the most common quartet topology found in the five trees constructed in the previous phase. The quartets were weighted by the square root of the proportion of the input trees that contained the majority quartet.

The results are shown in Table 6. Somewhat surprisingly, aggregating several inferred trees did not improve the accuracy, but the coverage was increased from 83% to 94%. The quartet accuracy remained roughly the same, while the Robinson-Foulds accuracy dropped. The quartet accuracy remained the same when the remaining taxa were forced into the tree by running the random walk and inserting the taxon at the first leaf that was reached. However, the Robinson-Foulds accuracy dropped further as a result of inserting those remaining taxa.

**Table 6:** Aggregating multiple trees

	**% taxa**	**R-F**	**Quartet**
	**inserted**	**Accuracy**	**Accuracy**
5 input trees (average)	83	62	85
output tree	94	48	85
output tree with forcing	100	39	83

The performance of the random walk algorithm as a supertree method. We generated 5 input trees by running the random walk five times independently on the COG840 alignment. We then ran the random walk algorithm with quartet queries evaluated by taking the induced quartet in each tree, and choosing the most common one. The guide tree was chosen as the subtree induced by 200 randomly chosen taxa on one of the 5 input trees.

One problem with using our algorithm for amalgamating trees on different taxa sets is that many quartets needed by our algorithm are not contained in all the input trees. For example, if all the input trees have coverage of 83%, the probability that an input tree contains a random quartet is roughly 0.83^4^≈0.5, meaning that most quartets will be decided based on just 2 or 3 votes, and some of them might not appear in any of the input trees, which means that another quartet query has to be asked. This problem will be more challenging for datasets where input trees have even lower coverage.

In the second experiment, we generated 100 bootstrap samples from the COG840 alignment and ran Neighbour Joining 100 times. We then ran the random walk algorithm with the majority vote quartet oracle, as in the previous experiment. The resulting tree contained over 98% of the taxa and had quartet accuracy of 94%, compared to the average accuracy of 88% for the input trees. The Robinson Foulds acccuracy dropped from 62% to 50%.

These experiments show that our random walk algorithm may be potentially useful in aggregating information from multiple trees. However, the trees obtained from the algorithm tend to sacrifice Robinson-Foulds accuracy to increase quartet accuracy or coverage, which may be considered a drawback of the method. If the input trees have low coverage, many quartets asked by the algorithm do not appear in any of the trees, which may harm speed and accuracy. We leave the solution to these two problems for future work.

## Conclusions

We have presented our work in progress to move our extremely fast quartet phylogeny algorithm from being a theoretical result to a practical algorithm for inferring trees. A variety of sensible heuristics, including weighting quartets by our confidence in them, using multiple quartet queries per insertion, and using multiple rounds of insertions, can increase both coverage and accuracy substantially over our original implementation. If we force all taxa into the tree and use local search, accuracy is comparable to existing programs. At present, our algorithm is close to being competitive with Neighbour Joining, while being much faster. For very large data sets, it may be suggested as one of few heuristics for the task.

There is still much future work: in particular, we are currently limited in part due to bad quartet inferences. If there were a way to bias our quartet choice toward high-quality quartets, this could substantially improve our quality. Also, it would be helpful if, after each round of insertions of taxa, we could remove taxa that are likely in a poor placement, and re-insert them. Finally, we are investigating a profiling technique, as described in the improvements section, to pre-identify a good starting search node for each new taxon, instead of the search tree root, where we predict errors are more common.

It remains to be seen whether a very fast quartet-based phylogeny method of our sort can in fact be competitive with the best algorithms. However, over the years, a number of other methods have grown up trading off speed with accuracy. We see evidence that that speed can be pushed to the theoretical limit, and that good trees can result from such procedures.

## Competing interests

The authors declare that they have no competing interests.

## Authors’ contributions

JT and DGB jointly conceived the approach. JT designed and implemented most of the algorithms and performed most of the experiments. YH implemented some of the algorithms and performed some of the experiments. DGB directed the project. All authors wrote parts of the manuscript. All authors read and approved the manuscript.
